# Extensive analysis of D7S486 in primary gastric cancer supports TESTIN as a candidate tumor suppressor gene

**DOI:** 10.1186/1476-4598-9-190

**Published:** 2010-07-13

**Authors:** Haiqing Ma, Desheng Weng, Yibing Chen, Wei Huang, Ke Pan, Hui Wang, Jiancong Sun, Qijing Wang, Zhiwei Zhou, Huiyun Wang, Jianchuan Xia

**Affiliations:** 1 State Key Laboratory of Oncology in Southern China and Department of Experimental Research, Sun Yat-sen University Cancer Center, 651 Dongfeng Road East, Guangzhou 510060, P. R. China; 2 Department of Gastric Pancreatic Surgery, Sun Yat-sen University Cancer Center, 651 Dongfeng Road East, Guangzhou 510060, P. R. China

## Abstract

**Background:**

High frequency of loss of heterozygosity (LOH) was found at D7S486 in primary gastric cancer (GC). And we found a high frequency of LOH region on 7q31 in primary GC from China, and identified D7S486 to be the most frequent LOH locus. This study was aimed to determine what genes were affected by the LOH and served as tumor suppressor genes (TSGs) in this region. Here, a high-throughput single nucleotide polymorphisms (SNPs) microarray fabricated in-house was used to analyze the LOH status around D7S486 on 7q31 in 75 patients with primary GC. Western blot, immunohistochemistry, and RT-PCR were used to assess the protein and mRNA expression of TESTIN (TES) in 50 and 140 primary GC samples, respectively. MTS assay was used to investigate the effect of TES overexpression on the proliferation of GC cell lines. Mutation and methylation analysis were performed to explore possible mechanisms of TES inactivation in GC.

**Results:**

LOH analysis discovered five candidate genes (*ST7*, *FOXP2*, *MDFIC*, *TES *and *CAV1*) whose frequencies of LOH were higher than 30%. However, only *TES *showed the potential to be a TSG associated with GC. Among 140 pairs of GC samples, decreased *TES *mRNA level was found in 96 (68.6%) tumor tissues when compared with matched non-tumor tissues (*p *< 0.001). Also, reduced TES protein level was detected in 36 (72.0%) of all 50 tumor tissues by Western blot (*p *= 0.001). In addition, immunohistochemical staining result was in agreement with that of RT-PCR and Western blot. Down regulation of TES was shown to be correlated with tumor differentiation (*p *= 0.035) and prognosis (*p *= 0.035, log-rank test). Its overexpression inhibited the growth of three GC cell lines. Hypermethylation of *TES *promoter was a frequent event in primary GC and GC cell lines. However, no specific gene mutation was observed in the coding region of the *TES *gene.

**Conclusions:**

Collectively, all results support the role of *TES *as a TSG in gastric carcinogenesis and that *TES *is inactivated primarily by LOH and CpG island methylation.

## Background

Gastric cancer (GC) is one of the leading causes of cancer mortality in the world, particularly in East Asian countries such as China, Japan and Korea, as well as other developing countries. Over the past decades, the overall survival for GC has not significantly improved in spite of improvement in surgical technique and significant advancement of chemotherapy and radiotherapy options [1]. Therefore, it is important to understand the molecular mechanisms involved in the carcinogenesis of GC.

Loss of heterozygosity (LOH) at specific sites of the cancer genome is considered to embody tumor suppressor genes (TSGs). Frequent LOH at 7q31.1/2 has been detected in many human malignancies including GC [2]. Recently, we found a high frequency of LOH region on 7q31 in primary GC from China, and identified D7S486 to be the most frequent LOH locus [3]. This study was designed to explore what TSGs associated with GC were located around D7S486 in this region.

Using microarray technology, a high-throughput single nucleotide polymorphisms (SNP) genotyping system was used to evaluate the LOH status around D7S486 on 7q31 in 75 primary GC samples and to discover possible candidate genes. As a result, *TESTIN *(*TES*) showed the potential to be a TSG in GC after initial screening. To clarify its role in GC, we examined TES expression in primary GC and its relationship to clinicopathological characteristics and prognosis. We also examined the effect of *TES *overexpression on the proliferation of several GC cell lines. Furthermore, mutation and methylation analysis were performed to explore its possible mechanisms of inactivation in GC.

## Results

### Identification of candidate tumor suppressor genes around D7S486 in primary GC

In this study, 75 pairs of DNA samples of tumor tissue and matched adjacent non-tumor tissue obtained by microdissection were extracted from patients with primary GC. For details of the result of microarray, please see Additional file 1 and Additional file 2.

**Figure 1:**
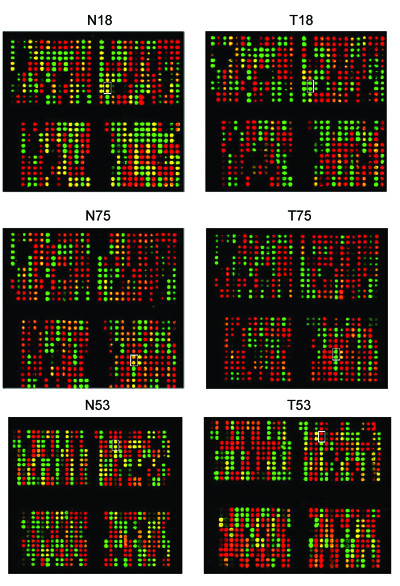
**Representative results of microarray images from 3 primary GC samples by microdissection. **"T" for tumor tissue and "N" for matched adjacent non-tumor tissue. (Each probe was printed twice and shown as neighboring spots. Spots in red and green, homozygous; yellow, heterozygous; and dark, low signal represented no signal or too low for genotype calls. White pane represented LOH, which is homozygous in tumor tissue but heterozygous in matched non-tumor tissue.)

Based on the NCBI database, there were total of 21 identified genes and 14 expressed sequence tags (EST) in the 4 Mb region (from 113 Mb to 117.5 Mb, please see figure S1, Additional file 3) around D7S486. 347 SNPs were selected in this region, including 254 SNPs located within genes and 93 SNPs located within ESTs. PCR primers and probes of these SNPs were designed by the previously described software [4]. After selection by pretesting amplification, 309 optimal primer pairs were determined (For ID of 309 SNPs, please see Additional file 4). Multiplex amplification was performed as described in the Methods section. After hybridization and genotype determination, 12 SNPs showed a frequency of LOH > 15%. Further, 6 of 12 SNPs displayed a frequency of LOH > 30% (Fig. 1 and Table 1) within 5 separate identified genes: *FOXP2*, *ST7*, *MDFIC*, *CAV1 *and *TES*. Among them, only *ST7 *[5], *CAV1 *[6] and *TES *[7-11] have been reported to be candidate tumor suppressor genes. However, little evidence supported *ST7 *and *CAV1 *to be tumor suppressor genes in primary GC. In addition, we used RT-PCR to detect the mRNA level of these candidate genes in 10 paired primary gastric cancer tissues and matched non-tumor tissues, respectively. And the results illustrated that *TES *mRNA expression was significantly down regulated in primary gastric cancer tissues than in non-tumor tissues, while other genes failed to show such a significant difference (please see figure S2, Additional file 5). Combining the above results in this paper with the evidence from previous studies, *TES *shows great potential to be a candidate TSG associated with GC.

**Table 1:** SNPs with frequency of LOH > 30% detected in 75 paired tumor tissue and matched adjacent normal tissue samples from GC patients

SNP ID	LOH	Informative Individuals	LOH (%)	Alleles	Allele Position/Total length	Heterozygosity (%)	Gene Link	Position in Gene
rs6980093	11	24	45.8	A/G	301/601	0.229	FOXP2	Intron
rs1881287	17	56	30.4	A/G	301/601	0.533	TES	Intron
rs2594458	9	27	33.3	A/G	301/601	0.257	MDFIC	Intron
rs193573	17	48	35.4	A/G	501/706	0.457	ST7	Intron
rs6466550	16	51	31.4	C/T	201/401	0.486	None	--
rs6466587	14	46	30.4	A/G	301/601	0.438	CAV1	Intron

### TES Expression in GC tissues

**Figure 2:**
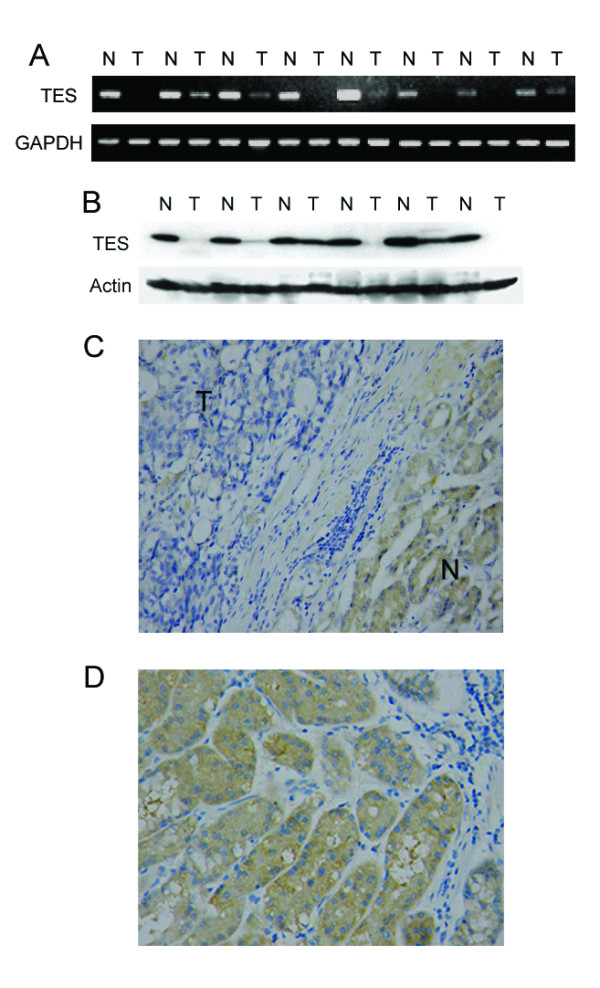
**TES expression in primary GC. **A. Representative results of *TES *mRNA expression in primary GC by RT-PCR. B. Representative results of TES protein expression in GC by Western blot. C. Representative staining of TES in GC tissue by immunohistochemistry (× 100). "T" and "N" represent tumor tissue and matched adjacent non-tumor tissue, respectively. D. Representative staining of TES in gastric non-tumor tissue (× 100).

Among 140 pairs of GC samples, decreased *TES *mRNA level was found in 96 (68.6%) tumor tissues when compared with matched non-tumor tissues (*p *< 0.001, Fig. 2A). Also, reduced TES protein level was detected in 36 (72.0%) of all 50 tumor tissues by western-blot in this study (*p *= 0.001, Fig. 2B). In addition, immunohistochemical staining result was in agreement with that of RT-PCR and Western blot. In all 140 paired GC samples, 32 (22.9%) cases displayed positive staining, primarily in the cytoplasm of the cells within the tumor tissues, while 120 (85.7%) cases exhibited positive staining in non-tumor tissues (*p *< 0.001, Fig. 2C). All of the results demonstrated a significant down regulation of TES mRNA and protein expression in primary GC.

### Relationship between TES protein expression and clinicopathological characteristics and prognosis in GC

As shown in Table 2, deletion or reduced TES protein expression was associated with tumor differentiation; the lower the differentiation, the lower TES expression. And a significant difference was found in the variance of TES positivity rates between the different groups (*p *= 0.007). By spearman analysis, the correlation value between TES expression and tumor differentiation was 0.178 (*p *= 0.035). However, TES expression failed to be correlated with age, sex, tumor size, metastasis, lymphoma invasion, T stage and clinical stage (*p *> 0.05).

**Table 2:** Relationship between TES protein expression and clinicopathological variables in 140 cases of primary GC

Clinicopathological variables	n^†^	TESTIN cases (%)		*p*-Value
			
		Positive	Negative	
Age (years)				0.384
<56	68	12 (17.5)	49 (80.3)	
≥56	72	17 (23.6)	55 (76.4)	
Gender				0.886
Female	45	9 (20.0)	36 (80.0)	
Male	95	20 (21.1)	75 (78.9)	
Tumor size (cm)				0.789
<5	61	12 (19.7)	49 (80.3)	
≥5	79	17 (21.6)	62 (78.4)	
T stage				0.441
T1	12	1 (8.3)	11 (91.7)	
T2	17	2 (11.8)	15 (88.2)	
T3	85	19 (22.4)	66 (77.6)	
T4	26	7 (26.9)	19 (73.1)	
N stage				0.520
N0	44	6 (13.6)	38 (86.4)	
N1	42	9 (21.4)	33 (78.6)	
N2	43	11 (25.6)	32 (74.4)	
N3	11	3 (27.3)	8 (72.7)	
M stage				0.997
M0	111	23 (20.7)	88 (79.3)	
M1	29	6 (20.7)	23 (79.3)	
Clinical stage				0.686
I	17	2 (11.8)	15 (88.2)	
II	28	5 (17.9)	23 (82.1)	
III	51	11 (21.6)	40 (78.4)	
IV	44	11 (25.0)	33 (75.0)	
Differentiation				0.007*
Well	3	2 (66.7)	1 (33.3)	
Moderate	30	12 (40.0)	18 (60.0)	
Poor	97	13 (13.4)	84 (86.6)	
Undifferentiated	10	2 (20.0)	8 (80.0)	

^† ^Number of cases in each group.

* Statistically significant (*p *< 0.05).

**Figure 3:**
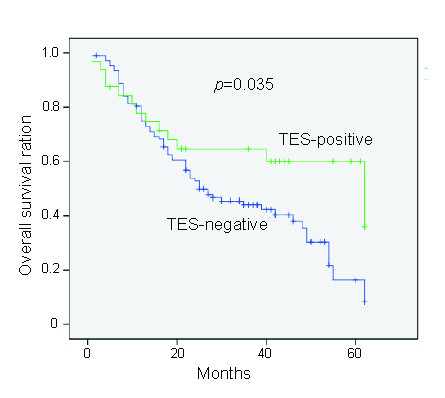
**Estimated overall survival according to the expression of TES in 140 cases of primary GC (Kaplan-Meier method). **The expression of TES was classified as negative expression (n = 108) and positive (n = 32) based on the immunohistochemical staining results.

In addition, the mean survival time of TES-positive patients was 42.5 ± 4.7 months and the median was 62.0 ± 13.1 months, while these two corresponding values were 32.6 ± 2.2 months and 25.0 ± 5.1 months in TES-negative patients, respectively. Using Kaplan-Meier curve assessment, we found that negative TES expression was a significant prognostic factor of poor overall survival in GC patients (*p *= 0.035, log-rank test), indicating that primary GC patients with positive TES expression showed a significantly longer survival time (Fig. 3).

### Overexpression of *TES *and its effect on proliferation of GC cells

**Figure 4:**
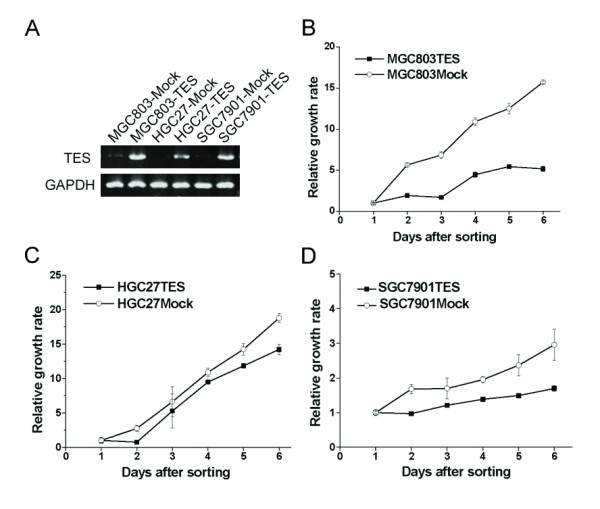
**Overexpression of *TES *and its inhibitory effect on the proliferation of GC cells. **Three GC cell lines used in this study were MGC803, HGC27 and SGC7901. A. TES mRNA expression in GC cells 36 h after transfection. B-D. The inhibitory effect of *TES *overexpression on the proliferation of GC cells sorted by flow cytometry for 7 consecutive days. This experiment is a representative of three independent experiments.

By RT-PCR, higher expression of *TES *was detected in all three cell lines transfected with pEGFP-C2/TES compared to the cell lines transfected with the empty pEGFP-C2 plasmid (Fig. 4A). MTS proliferation assay showed that relative growth rate in all three pEGFP-C2/TES-transfected cells were significantly lower than those counterparts in pEGFP-C2-transfected cells (*p *< 0.01) (Fig. 4B, C, D).

### *TES *gene mutation analysis

To detect mutations of the *TES *gene, direct sequencing of all 7 exons of the *TES *gene was performed in three gastric cell lines and five cases of GC with rs1881287 LOH. No mutation was detected in exons 1, 2, 3 and 5. However, in the HGC27 cell line, we detected a heterozygous nucleotide change in codon 221 of *TES *transcript 1 in exon 4, from GCC to GTC, with an amino acid substitution from alanine to valine. We also detected a heterozygous nucleotide change in codon 364 in exon 7 of HGC27 and SGC7901 cells, from CGG to CGC, both encoding arginine, and a homozygous change from CGG to CGC at the same codon in Case 60. We searched the NCBI SNP database and found that all of the above mutation sites are in accordance with the cSNP sites of the *TES *gene. Another heterozygous point mutation was detected in codon 312 of exon 6 from Case 61, from GAG to GGG, with the amino acid substitution from glutamic acid to glycine, but the non-tumor tissue from the same case also showed the same heterozygous point mutation. While the other non-tumor counterparts did not show any evidence of mutation.

### Abnormal DNA methylation of *TES *promoter in GC

**Figure 5:**
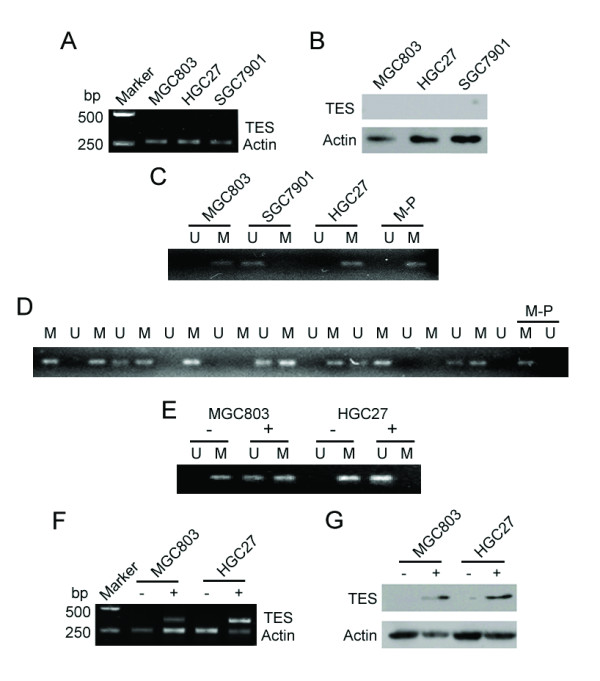
**DNA Methylation analysis of *TES *promoter in GC. **TES mRNA (A) and protein (B) expression were significantly down regulated in three GC cell lines (MGC803, HGC27 and SGC7901). Methylation analysis of TES promoter in three GC cell lines (C) and in 10 pairs of GC samples (D). Complete methylation was shown in MGC803 and HGC27 cells and in most GC samples. After DAC treatment, the complete methylation of *TES *promoter changed to partial methylation and nonmethylation in MGC803 and HGC27 cells, respectively (E). DAC treatment also reversed *TES *mRNA (F) and protein (G) expression in MGC803 and HGC27 cells. "M", "U" and"M-P"represent methylated CPG sequences, unmethylated CPG sequences and a positive control for methylation, respectively. "-" and "+" represent before and after DAC treatment, respectively.

The mRNA and protein expressions of TES in MGC803, HGC27 and SGC7901 GC cell lines were not detected by RT-PCR and Western blot, respectively (Fig. 5A, B). Methylation analysis revealed complete methylation in MGC803 and HGC27 cells, while no methylation was detected in SGC7901 cells (Fig. 5C). In addition, 30 primary GC samples without TES expression as determined by immunohistochemistry were selected for methylation analysis in the promoter of *TES *gene. Among them, 18 samples showed complete methylation in the *TES *promoter, 4 with partial methylation and 8 were unmethylated (Fig. 5D).

5-Aza-2'-deoxycytidine (decitabine; DAC) is a pyrimidine nucleoside analog that strongly inhibits DNA methyltransferase activity and, as such, is one of the strongest inhibitors of DNA methylation and is also an effective antileukemic agent [12]. Since the GC cell lines MGC803 and HGC27 displayed complete methylation in the *TES *promoter, we further investigated the effect of DAC on its methylation. Interesting, after treated with DAC, the complete methylation status in the *TES *promoter changed to partial methylation and nonmethylation in MGC803 and HGC27 cells, respectively (Fig. 5E). In addition, mRNA and protein level of TES also changed from negative to positive in both MGC803 and HGC27 cells after DAC treatment (Fig. 5F, G) as determined by RT-PCR and Western blot, respectively.

## Discussion

In our previous studies [3,13,14], we found that the frequency of LOH at D7S486 was as high as 30.36% in primary GC tissues. We also found that most GC patients with a high frequency of LOH at D7S486 were in their middle to advanced clinical stage (stage III and IV), and usually correlated with lymph node metastasis [3,13,14]. Taken together, these results suggest that one or more tumor suppressor genes associated with gastric carcinomas might situate around D7S486.

In this study, using a high-throughput SNPs microarray fabricated in-house, we performed the SNP LOH assay in 75 paired GC samples. These SNPs are located within genes and ESTs around D7S486 and with a heterozygosity frequency greater than 0.1. As a result, 6 SNPs with a LOH frequency of > 30% were identified. After bioinformatics analysis, 5 corresponding identified genes were determined, including *ST7*, *FOXP2*, *MDFIC*, *TES *and *CAV1*. Among these genes, no reports have shown any correlations between *FOXP2 *and *MDFIC *and human malignancy. Although *ST7 *[5] and *CAV1 *[6] have been reported to be candidate tumor suppressor in several types of cancer, no direct evidences support their relationship with gastric carcinogenesis [5,14-16]. However, many previous studies on *TES *[7-11,17] have demonstrated that it may be a candidate TSG in human cancers.

Human gene encoding TES was first identified by Tatarelli et al. [9] and localized to the fragile site FRA7G at 7q31.2. So far, down regulation of TES has been reported in many types of human malignancies [8,10]. In addition, a profound reduction in growth potential was detected in different cancer cell lines in which *TES *was overexpressed [9,11]. All these findings suggest that *TES *may present a candidate tumor suppressor gene. However, to date, there is no definitive report regarding whether *TES *works as a TSG in primary GC.

One of the most notable features of TSG is the mutation that may lead to gene inactivation, especially mutation in exon. Missense mutation has been detected in exons 3 and 5 of *TES *in the leukemic cell line CEM and in the ovarian cancer cell line CAOV3 [10]. However, no mutation was detected in these two exons in our study. A heterozygous mutation at codon 221 in exon 4 of *TES *has been reported in the breast cancer cell line MDA-MB 453 [9] and in head and neck squamous cell carcinoma tissues [8]. Similarly, the same mutation in exon 4 was also detected in HGC27 GC cells in this study. And a sense point mutation was also found in exon 7 of *TES*. However, all the above substitutions were in accordance with the cSNP sites of the *TES *gene, which had already been recorded in the NCBI SNP database, suggesting that they were not tumor-specific. Moreover, in exon 6, we found a new heterozygous missense point mutation in a GC tissue sample. Although it has not been reported, the same mutation was also observed in the matched non-tumor tissue. The above findings suggest that gene mutations may not be a mechanism for *TES *inactivation in GC.

When LOH occurs in a gene, the expression of the gene may be reduced. Therefore, we then investigated the expression of *TES *in primary GC. As expected, both transcriptional and translational level of TES were reduced in primary GC tissues, which is consistent with its expression level in other types of human tumors, indicating that the deletion or down regulation of *TES *might be involved in the occurrence and development of GC. Furthermore, less differentiated tumors exhibited lower TES expression, indicating a significant correlation between TES expression and tumor differentiation. In addition, patients with negative expression of TES had a shorter life span than those with positive expression, suggesting that the detection of TES expression might be helpful to assess prognosis in GC. In this study, we also found for the first time that the introduction of *TES *caused a significant growth delay in three GC cell lines. This result demonstrated the suppressor effect of *TES *on GC cells, supporting the idea that *TES *functions as a tumor suppressor gene in GC, at least in these three cell lines.

However, the mechanism of down regulation of TES expression in GC is not fully understood. Besides LOH causing reduction of gene expression, hypermethylation of CpG islands located in the promoter region of a gene is also frequently correlated with its transcriptional down regulation [18], and represents an alternative mechanism for the inactivation of TSG [19]. Several teams have reported on the methylation status of *TES *promoter in different tumor types. Tobias [11] found frequent methylation of the CpG islands at the 5'end of *TES *in 7 of 10 ovarian carcinomas and in all 30 tumor-derived cell lines tested, but only discovered a frame-shift mutation in one allele of a breast cancer cell line and polymorphisms in a number of samples. Tatarelli [10] found the *TES *promoter was fully methylated in the majority of 46 tumor-derived cell lines, and only 3 of 46 tumor-derived cell lines showed partial methylation at the promoter of *TES *gene. Similarly, no homozygous deletions or a high frequency of convincing somatic mutations was found within the coding region of *TES *in a total of 26 cancer cell lines. All of these data demonstrate that *TES *is inactivated primarily by transcriptional silencing resulting from CpG island methylation rather than gene mutation.

In this study, looking at 30 primary GC samples with reduced TES expression as determined by immunochemistry, 18 samples were found to be completely methylated at the promoter of *TES*, 4 partially methylated and 8 were found to be unmethylated. In three GC cell lines with an absence of TES expression, the MGC803 and HGC27 cells displayed complete methylation at the *TES *promoter, while no methylation was detected in SGC7901 cells. These results suggest that the methylation of CpG in the *TES *promoter was a frequent event in GC and might be involved in the inactivation of TES.

In this study, we further investigated the effect of DAC, a pyrimidine nucleoside analog that strongly inhibits DNA methyltransferase activity by titrating out DNA methyltransferase activity via a covalent trapping mechanism, on MGC803 and HGC27 GC cell lines, which showed complete methylation at the *TES *promoter. Interestingly, after DAC treatment, the complete methylation of *TES *promoter changed to partial methylation and nonmethylation in MGC803 and HGC27 cells, respectively. The mRNA and protein level of TES also changed from negative to positive in both MGC803 and HGC27 cells following DAC treatment. These findings further confirm that methylation of *TES *promoter plays an important role in TES down regulation in GC.

In conclusion, the present study provides novel and definitive data to support the idea that *TES *may play an important role in primary GC as a tumor suppressor, and also conforms that, besides LOH, hypermethylation in the promoter of the *TES *gene rather than gene mutations contributes to its inactivation and to the progression of gastric carcinogenesis.

## Methods

### Loss of heterozygosity analysis of the genes around D7S486 by SNP genotyping system

#### Patients and tissue specimens

75 paired fresh GC and matched adjacent non-tumor tissue specimens were obtained from patients underwent surgical resection but without any preoperative treatment in the Sun Yat-sen University Cancer Center between 2004 and 2005. The 75 patients included 54 males and 21 females with a median age of 60 years. After surgical resection, the fresh tissues were immediately immersed in RNAlater (Ambion, USA) and stored at 4°C overnight to allow thorough penetration of the tissues, then frozen at -80°C until RNA and DNA extraction. Both cancer and matched adjacent non-tumor tissues not less than 2 cm away from the GC patients were sampled, respectively, and confirmed by pathological examination. The study was approved by the Ethics Committee of Sun Yat-sen University Cancer Center and informed consent was obtained from each patient.

#### DNA extraction from microdissected GC tissue

DNA samples were extracted from 75 paired GC specimens obtained by microdissection using TRIzol kit (Invitrogen, Carlsbad, CA, USA) according to the manufacturer's protocol. The procedures for microdissection were performed as previously described [20].

#### SNP genotyping and LOH analysis by microarray system

##### SNP Selection

A computer program written for SNP selection was used as previously described [4]. SNPs in a 4 Mb region around D7S486 on human chromosome 7q were selected from the dbSNP database ftp://ftp.ncbi.nih.gov/snp/organisms/human_9606/chr_rpts/ maintained by National Center for Biotechnology Information (NCBI, Build 36.3). To ensure that the selected SNPs were real and suitable for the multiplex system, a series of criteria described before was used for selection [21].

##### Design of primer and probe

To select sequence frames for primers, a computer program described [4] before was used. Within a user-defined sequence range around the polymorphic sites (150 bp in the present study), the candidate sequence frames were first selected according to a user-defined melting temperature range (55°C to 75°C in this application). To minimize primer-primer interactions, further selection was performed on qualified frames based on a series of criteria described before [4].

##### Multiplex PCR and ssDNA preparation

The procedures for multiplex amplification were performed following the method of Wang *et al*. [4] with minor modifications. In brief, the first multiplex PCR reaction was performed in 25 μl of PCR mix containing 2.5 μl of 10 × PCR buffer (50 mM KCl, 100 mM Tris-HCl at pH 8.3, 1.5 mM MgCl_2_, and 100 μg/ml gelatin), 0.5 μl of 10 nM dNTPs, primer mix (300 nM each) for all SNPs in the multiplex group, 5 units of HotStart *Taq *DNA polymerase, and 200 ng of DNA. The PCR cycling conditions were: 95°C (15 min) for 1 cycle, 94°C (40 sec), 55°C (2 min), ramping from 55°C to 72°C (5 min) for 40 cycles and a final extension of 72°C (10 min). ssDNA was generated in both directions using the same conditions for multiplex PCR with the following modifications: (1) 1.0 μl of product from the multiplex PCR was used as templates, (2) only one primer (one of the primer-probes) for each SNP was used, and (3) 45 PCR cycles were performed.

##### SNP genotype determination by microarray

In the SNP genotyping system [4] adopted in this paper, after generating single-stranded DNAs (ssDNA), they were hybridized to the probes on a microarray fabricated in-house. The probes were designed in such a way that their 3' ends were adjacent to the polymorphic sites in the hybridizing ssDNA. In this way, the probes could be labeled with the commonly used single-base extension method [22-24], during which single dideoxyribonucleotides (ddNTPs) were added to the probe in an allele-specific manner that was dependent on the hybridizing allelic sequence(s). When the corresponding ddNTPs were labeled with different fluorescent chromophores (cyanine dyes, either Cy3 or C5, in our system), the allelic state of the SNPs were determined by analyzing the amount of incorporated fluorescence with the written program "AccTyping" [25]. In addition, SNP genotypes could be determined independently with the two DNA strands as separate templates so that results from such dual-probe analysis could be compared to ensure a high degree of accuracy.

### Expression of TES in primary GC samples and its relationship to clinicopathological characteristics

140 paraffin-embedded GC samples were retrieved according to the 1999-2001 surgical pathology files in the Sun Yat-sen University Cancer Center, which included the patients without pretreatment. The 140 patients included 95 males and 45 females with a median age of 56 years. All tissue blocks were cut into serial 4 μm thick sections. The histological types were assigned according to WHO classification criteria.

Total RNA was extracted from 140 patients with primary GC using TRIzol solution (Invitrogen, Carlsbad, CA, USA) according to the manufacturer's protocol, and RNAse-free DNase I was used to remove DNA contamination. Reverse transcription (RT) was performed with 2 μg total RNA using M-MLV Reverse transcriptase (Promega, Madison, WI, USA) to synthesize first-strand cDNA according to the manufacturer's recommendation, followed by cDNA amplification using the specific primer set for *TES *and the GAPDH primer set used as an internal control. Primers used in this study were as follows: 5'-ACTGTGGCAGACATTACTGTGACA-3' (sense) and 5'-GATAGCTATGGCTCGATACTTCTGGGTGC-3' (antisense) for *TES*; 5'-CGGGAAGCTTGTCATCAATGG-3' (sense) and 5'-G GCAGTGATGGCATGGACTG-3' (antisense) for *GAPDH*, and the corresponding PCR products were 440 bp and 358 bp, respectively.

Western blot and immunohistochemistry analysis was carried out as our previously described [16]. Primary polyclonal antibody against TES (Santa Cruz, CA, USA) was used at 1:500 and 1:100 dilutions for Western blot and immunohistochemistry, respectively. As our previously described [16], the total TES immunohistochemical staining score was calculated as the sum of the percent positivity of stained tumor cells and the staining intensity.

### Overexpression of *TES *and its effect on proliferation of GC cells

Three GC cell lines used in this study were MGC803, SGC7901 and HGC27. All cell lines were obtained from the Committee of Type Culture Collection of Chinese Academy of Sciences (Shanghai, China) and preserved in our lab, and grown in RPMI 1640 supplemented with 10% FBS (fetal bovine serum) and antibiotics (50 μg/ml each of penicillin, streptomycin and gentamicin) at 37°C in a humidified 5% CO_2 _atmosphere.

Plasmid pOTB7 containing full-length *TES *cDNA were purchased from Invitrogen (Carlsbad, CA, USA). The *TES *cDNA was amplified with the following primers: upstream 5'-GCAAGCTTCATGGACCTGGAAAAC-3', downstream 5'-GCGGATCCCTAAGACATCCTCTT-3'. The 1283 bp full length *TES *cDNA PCR product was recovered with the QIAquick Gel Extraction Kit (Qiagen Inc., Valencia, CA). The *TES *cDNA was first cloned into a pGEM-T and then into a pEGFP-C2 vector to create the pEGFP-C2/TES eukaryotic expressing vector in which the TES cDNA was fused with the downstream of EGFP cDNA.

Three gastric cell lines were transfected with pEGFP-C2/TES or empty pEGFP-C2 plasmid, respectively, using LipofectamineTM 2000 reagent (Invitrogen, Carlsbad, CA, USA) according to the manufacturer's instructions. Briefly, cells were seeded into a 75 cm^2 ^culture flask, and 12-24 μg plasmid and 36-60 μl Lipofectamine 2000 reagent were used for transfection when cells were at 90% confluence. Cells were harvested 36 h after transfection and tested in the proliferation assay or harvested for RT-PCR detection.

Measurement of proliferation was determined by the MTS assay. Briefly, GFP-positive cells were sorted 36 h after transfection with BD Aria II(BD) and then seeded into 96-well plates (1000 cells per well) in 100 μl RMPI 1640 medium for five replicates. The next day, after 20 μl CellTiter 96 Aqueous One Solution (Promega, Madison, WI, USA) were added into the medium, the cells were incubated at 37°C for 4 h, and the absorbance at 490 nm (OD490) was measured daily for 7 days.

### Mutation analysis of *TES *gene

Each exon of *TES *was PCR amplified respectively. The PCR mixture contained 1 μg genomic DNA, 20 μl 2 × Pfu PCR MasterMix (TIANGEN Biotech, Beijing, China) and 0.1 pmol/μl of each primer in a total 40 μl volume. The PCR cycling conditions for exons 2 to 7 were: 94°C (2 min) for 1 cycle, 94°C (30 sec), 55°C (40 sec), 72°C (2 min) for 40 cycles and a final extension of 72°C (10 min). Exon 1 PCR conditions were as described above with the exception that the initial denaturation was performed at 97.6°C for 5 min, and the denaturation temperature in the 40 cycles was also set at 97.6°C. For identification of mutations, PCR products were purified and then sequenced on an ABI Prism 3700 automated DNA Analyzer. All mutations were confirmed by sequencing in both directions.

### Methylation analysis of *TES *promoter in primary GC

Three GC cell lines used in this study were MGC803, SGC7901 and HGC27 with the same culture condition as described above. 10 μM of 5-aza-2'-deoxycytidine (decitabine; DAC) was added when cell density reached 75%. Culture medium was changed with fresh RPMI1640 with the same concentration of DAC on alternate days. PBS (10 μM) was used as a negative control. Cells were harvested for the experiment six days later.

The genomic DNA isolated with the DNeasy Tissue Kit (Qiagen Inc., Valencia, CA) was modified by bisulfite treatment with the CpGenome DNA Modification Kit (Chemicon) and amplified by PCR with two sets of *TES *promoter specific primer pairs (M-*TES *sense 5'-TATTGAGTTTGTTTAGTAGGGCGTC-3', M-*TES *antisense 5'-AATAACAACCGAACAACTCCG-3', PCR product 133 bp; U-*TES *sense 5'-TGAGTTTGTTTAGTAGGGTGTTG-3', U-*TES *antisense 5'-ATAACAACCAAACAACTCCAA-3', PCR product 129 bp) that recognize either the methylated or unmethylated CpG sequences and then analyzed by electrophoresis.

### Statistical analysis

Quantitative values were expressed as the means ± SD or median (range). The Quantity One software (Bio-Rad Laboratories, Inc. Hercules, CA, USA) was used to quantify the densities of the bands in RT-PCR and Western blot. Paired-samples t-test was used to compare mRNA and protein expression of TES in GC with paired adjacent non-tumor tissue samples. The χ^2 ^test for proportion and Spearman's correlation was used to analyze the relationship between TES expression and various clinicopathological characteristics. Survival curves were calculated using the Kaplan-Meier method and compared by the log-rank test. The SPSS 15.0 software (SPSS Inc., Chicago, IL, USA) was used for all statistical analyses and *p *< 0.05 was considered significant.

## Competing interests

The authors declare that they have no competing interests.

## Authors' contributions

DW, HM, YC, WH, and KP participated in the acquisition of data. ZZ, HW, and JX were involved with the study concept and design. DW, HM, JS, HW, and QW contributed to the statistical analysis. HM, DW, JX participated in manuscript preparation. All authors participated in the interpretation of results and critical revision of the manuscript for important intellectual content. All authors have read and approved the final manuscript.

## Supplementary Material

Additional file 1 **microarray data**. SNP microarray result from 75 patients with primary GC. Click here for file

Additional file 2 **Patient information**. Information of 75 patients in SNP microarray assay. Click here for file

Additional file 3 **Figure S1 - Schematic figure**. Schematic figure of the 4 Mb region (from 113 Mb to 117.5 Mb) around D7S486. Click here for file

Additional file 4 **SNP ID**. ID of 309 SNPs in microarray assay. Click here for file

Additional file 5 **Figure S2 - mRNA expression of 5 genes in primary GC**. Representative result of the mRNA expression levels of 5 genes, FOXP2, MDFIC, ST7, CAV1 and TES in primary GC by RT-PCR. "T" and "N" represent tumor tissue and matched adjacent non-tumor t from patients with primary GC issue, respectively. Click here for file
