# Supplementary figures and images for: Extensive analysis of D7S486 in primary gastric cancer supports TESTIN as a candidate tumor suppressor gene
Source: Mol Cancer. 2010 Jul 13;9:190. doi: 10.1186/1476-4598-9-190 (PMC2915979; doi:10.1186/1476-4598-9-190)

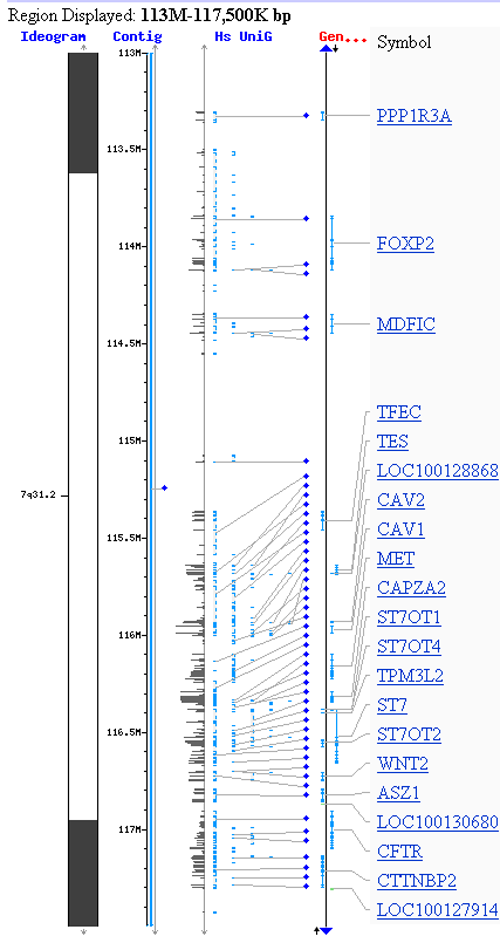

Supplement: Additional file 3 — Figure S1 - Schematic figure. Schematic figure of the 4 Mb region (from 113 Mb to 117.5 Mb) around D7S486. [file 1476-4598-9-190-S3.TIFF]

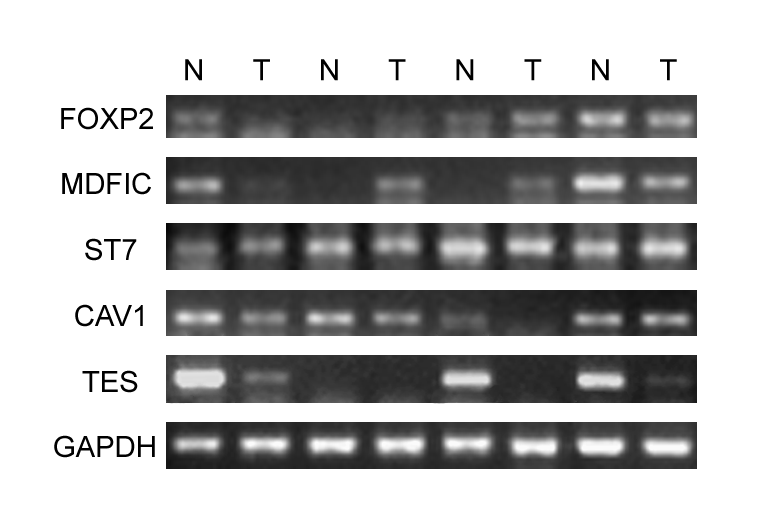

Supplement: Additional file 5 — Figure S2 - mRNA expression of 5 genes in primary GC. Representative result of the mRNA expression levels of 5 genes, FOXP2, MDFIC, ST7, CAV1 and TES in primary GC by RT-PCR. "T" and "N" represent tumor tissue and matched adjacent non-tumor t from patients with primary GC issue, respectively. [file 1476-4598-9-190-S5.TIFF]
